# A general model for designing the chirality of exciton-polaritons

**DOI:** 10.1515/nanoph-2024-0662

**Published:** 2025-02-03

**Authors:** Ping Bai, Siying Peng

**Affiliations:** School of Engineering, 557712 Westlake University, Hangzhou, Zhejiang 310030, China; Research Center for Industries of the Future and School of Engineering, 557712 Westlake University, Hangzhou, Zhejiang 310030, China

**Keywords:** chirality, exciton-polaritons, degree of circular polarization

## Abstract

Chirality of exciton-polaritons can be tuned by the chirality of photons, excitons, and their coupling strength. In this work, we propose a general analytical model based on coupled harmonic oscillators to describe the chirality of exciton-polaritons. Our model predicts the degree of circular polarization (DCP) of exciton-polaritons, which is determined by the DCPs and weight fractions of the constituent excitons and photons. At the anticrossing point, the DCP of exciton-polaritons is equally contributed from both constituents. Away from the anticrossing point, the DCP of exciton-polaritons relaxes toward the DCP of the dominant constituent, with the relaxation rate decreasing as the coupling strength increases. We validate our model through simulations of strongly coupled topological edge states and excitons, showing good agreement with model predictions. Our model provides a valuable tool for designing the chirality of strong coupling systems and offers a framework for the inverse design of exciton-polaritons with tailored chirality.

## 1 Introduction

Exciton-polaritons are quasi-particles that are half-light half-matter, arising from the strong coupling of photons in optical microcavities and excitons in semiconductor materials. Due to their photonic component, exciton-polaritons exhibit propagation characteristics that can be described by a well-defined dispersion relation [1], [2]; their excitonic component, on the other hand, imparts stronger nonlinear behavior compared to pure photons [3], [4], [5]. The generation of exciton-polaritons facilitates the modulation of the spontaneous emission rate [6], [7], spectral linewidth [8], and polarization characteristics of emitting materials [9], [10]. Investigating the chiral emission of exciton-polaritons is essential for gaining deeper insights into quantum optical phenomena in strong light–matter coupling, serving as a foundation for the development of spin-based active optical devices and tunable optoelectronic systems. This research uncovers new physical phenomena, such as quantum entanglement and chiral quantum light fields [11], while also offering practical implications for the advancement of cutting-edge optoelectronic devices and quantum information technologies. Chiral exciton-polaritons, for instance, hold potential for realizing all-optical switches [12], [13], [14], optical logic gates [15], novel quantum light sources [16], [17], and optical isolators [18], furthering advancements in quantum computing and communication.

The chirality of exciton-polaritons can originate from asymmetric chiral semiconductor materials [19], [20], [21] or from chiral optical modes in the microcavities [22], [23], [24], [25], [26], [27], [28]. The chirality of semiconductor materials refers to their structural property of being different from their mirror image [29], [30], [31]. This asymmetry results in different responses to circularly polarized light, such as left- or right-handed polarization. Chiral materials are important in many fields, including optics, pharmaceuticals, biomolecules, and nanotechnology, as their physical, chemical, and biological properties depend on their chiral form. Chiral microcavities are specially designed optical cavities that can differentiate and enhance the circular polarization states of light (left- or right-handed). These cavities control and manipulate the chirality of light through their chiral structural properties [24], [32], [33], [34], [35], resulting in optical modes with specific handedness. Chiral microcavities have applications in chiral molecule detection [36], [37], [38], quantum information processing [39], and optical signal isolation [40], [41].

In this work, we propose a general analytical model to investigate the mechanisms underlying chirality of exciton-polaritons in strong light–matter coupling systems, initiated from the coupled harmonic oscillator model. The proposed model determines the DCP of exciton-polaritons and introduces a new criterion for chiral strong coupling upon their formation. Specifically, the model addresses three key behaviors: (a) At the anticrossing point, the DCP of the exciton-polaritons is equally derived from the DCPs of both exciton and photon. (b) Away from the anticrossing point, the DCP of exciton-polaritons gradually shifts toward the dominant constituent (either exciton or photon), with the rate of change decreasing as the coupling strength increases. (c) The same exciton-polariton band is expected to detect a full range of DCP values from left-handed to right-handed luminescence simultaneously.

To validate the model, we simulate a photonic crystal superlattice with topological edge states, which are strongly coupled with excitons in 2D halide perovskites. Our results show that the chiral response of the topological edge states is transferred to the exciton-polariton bands. The simulated DCP for exciton-polaritons agrees closely with predictions from our analytical model. Our study provides the understanding of chiral inheritance in exciton-polaritons, and our analytical model enables the inverse design of exciton-polaritons with specific chirality.

## 2 Theoretical model for the DCP of exciton-polaritons

We commence our theoretical analysis by investigating the strong light–matter interaction between a single exciton in the material and a photon state confined within a microcavity. This interaction can be modeled using a two coupled harmonic oscillator Hamiltonian, expressed as:

(1) $$H=\left[\begin{array}{cc} E_{e}-i\frac{\gamma_{e}}{2} & g\\ g & E_{p}-i\frac{\gamma_{p}}{2} \end{array}\right],$$

where *E*_*e*_ and *E*_*p*_ correspond to the energies of the exciton and cavity photon, respectively, and *γ*_*e*_ and *γ*_*p*_ denote their decay rates, given by the full-width at half-maximum (FWHM). The coupling strength *g*, which represents the coherent interaction between the excitons and photons, is determined by the exciton’s dipole moment *μ*, the electric field direction of the cavity mode *u*_*e*_, the number of excitons *N*, and the cavity mode volume *V*. It is expressed as: $g=\mu \cdot u_{e}\sqrt{\frac{NE_{p}}{\epsilon_{0}V}}$ . The energies of the lower and upper polariton bands for zero detuning are given by:

(2) $$E_{\text{UP/LP}}=E_{0}-\frac{i\left(\gamma_{e}+\gamma_{p}\right)}{4}\pm \frac{1}{2}\sqrt{{\left(2g\right)}^{2}-\frac{{\left(\gamma_{e}-\gamma_{p}\right)}^{2}}{4}}.$$

By tuning *g*, we can distinguish the energies and DCP of chiral exciton-polaritons in strong coupling chiral cavities [42], [43].

The eigenvectors and eigenvalues of the system can be determined by diagonalizing the Hamiltonian matrix. This process is represented as:

(3) $$H=MEM^{-1},$$

where *E* is a diagonal matrix containing the eigenvalues, which correspond to the exciton-polariton energies,

(4) $$E=\left[\begin{array}{cc} E_{\text{LP}} & 0\\ 0 & E_{\text{UP}} \end{array}\right].$$

Here, *E*_LP_ and *E*_UP_ represent the energies of the lower and upper polaritions, respectively, arising from the splitting at zero detuning (*E*_*e*_ = *E*_*p*_), with the energy difference given by the Rabi splitting (*ℏ*Ω). The matrix *M* consists of the eigenvectors of *H*, which are the Hopfield coefficients, i.e.,

(5) $$M=\left[\begin{array}{cc} \alpha_{1} & \alpha_{2}\\ \beta_{1} & \beta_{2} \end{array}\right].$$

These coefficients describe the weight fractions of exciton and photon in the hybrid exciton-polaritons. In the lower polariton (LP) branch, the exciton and photon contributions are |*α*_1_|^2^ and |*β*_1_|^2^, respectively, with the normalization condition |*α*_1_|^2^ + |*β*_1_|^2^ = 1. Similarly, in the upper polariton (UP) branch, the exciton and photon contributions are |*α*_2_|^2^ and |*β*_2_|^2^, satisfying |*α*_2_|^2^ + |*β*_2_|^2^ = 1.

For both chiral excitons and chiral photons, the different response to LCP and RCP light can be quantified by the DCP, which is defined as:

(6) $$\rho_{c}=\left(I_{\text{LCP}}-I_{\text{RCP}}\right)/\left(I_{\text{LCP}}+I_{\text{RCP}}\right),$$

where *I*_LCP_ and *I*_RCP_ represent the intensities of LCP and RCP light, respectively. For excitons, these intensities include the absorption or emission of circularly polarized light [44], [45], as well as the transmission or reflection of LCP and RCP waves in a chiral medium with a polarization-dependent refractive index [27], [46]. For cavity photons, the intensities relate to the propagation of LCP and RCP light. The DCP is defined to distinguish between LCP and RCP components.

In a strong coupling system with chiral exciton-polaritons, both chiral exciton and chiral photon are involved. We define the DCP of the uncoupled exciton and photon as $\rho_{c}^{e}$ and $\rho_{c}^{p}$ , respectively, which will be inherited into the exciton-polaritons with their respective weight fractions. Therefore, the DCPs of the LP and UP modes can be expressed as:

(7) $$\rho_{c}^{\text{LP}}=\rho_{c}^{e}\times |\alpha_{1}{|}^{2}+\rho_{c}^{p}\times |\beta_{1}{|}^{2},$$

(8) $$\rho_{c}^{\text{UP}}=\rho_{c}^{e}\times |\alpha_{2}{|}^{2}+\rho_{c}^{p}\times |\beta_{2}{|}^{2},$$

where |*α*_1_|^2^ and |*β*_1_|^2^ represent the weight fractions of the exciton and photon in the LP mode, while |*α*_2_|^2^ and |*β*_2_|^2^ represent the respective weight fractions in the UP mode. These values described in matrix *M* in Eq. (5) are calculated by diagonalizing the Hamiltonian.

## 3 Analytical model predictions of DCP in exciton-polaritons

**Figure 1:**
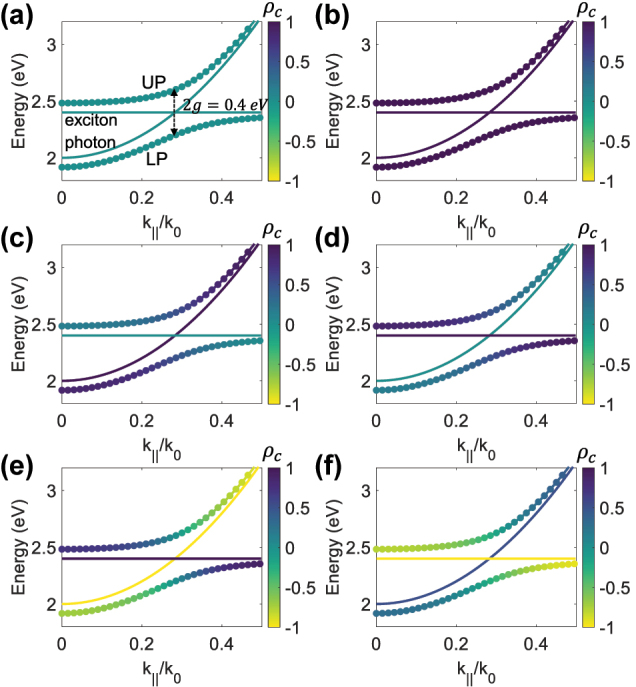
(color online). Analytical model predictions of the energies of the lower and upper polariton bands in dispersion, with their DCP values *ρ*_*c*_ color coded into the bands. The following assumptions are made: (a) $\rho_{c}^{e}=0,\;\rho_{c}^{p}=0$ , (b) $\rho_{c}^{e}=1,\;\rho_{c}^{p}=1$ , (c) $\rho_{c}^{e}=0,\;\rho_{c}^{p}=1$ , (d) $\rho_{c}^{e}=1,\;\rho_{c}^{p}=0$ , (e) $\rho_{c}^{e}=-1,\;\rho_{c}^{p}=1$ , (f) $\rho_{c}^{e}=-1,\;\rho_{c}^{p}=0.5$ . $\rho_{c}^{e}$ and $\rho_{c}^{p}$ are color coded into the exciton energy (*E*_*e*_ = 2.4 eV) lines and the parabolic photon curves. The coupling strength is set *g* = 0.2 eV.

To demonstrate the DCP of exciton-polaritons in a strong coupling system, we assume a parabolic dispersion for the chiral photon mode in a cavity, with photon energy defined as $E_{p}={\left(k_{\|}/k_{0}\right)}^{2}/0.2+2$ , where *k*_‖_/*k*_0_ ranges from 0 to 0.5. The exciton energy is *E*_*e*_ = 2.4 eV. For *γ*_*e*_ = 0, *γ*_*p*_ = 0, and *g* = 0.2 eV, we show the polariton energies and their DCP in Figure 1. When both the exciton and photon are achiral ( $\rho_{c}^{e}=0$ and $\rho_{c}^{p}=0$ ), the hybrid LP and UP modes are also achiral, as shown in Figure 1(a). Similarly, when both are fully LCP ( $\rho_{c}^{e}=1$ and $\rho_{c}^{p}=1$ ), the LP and UP modes are fully LCP, as shown in Figure 1(b). These cases confirm the validity of the model.

In Figure 1(c) and (d), we show the DCPs of exciton-polaritons for strong coupling between an achiral exciton and a fully LCP photon, and between a fully LCP exciton and an achiral photon. The DCP of the polaritons varies from 0 to 1, reaching 0.5 at zero detuning, indicating an LCP and RCP intensity ratio of 3:1 for both LP and UP modes. As the system deviates from the anticrossing point, the DCP of the polaritons gradually returns to the DCP of the exciton or photon, and their energies move closer to those of the exciton or photon.

In Figure 1(e) and (f), we consider cases where the chirality of the exciton and photon is opposite. For example, in Figure 1(e), a fully LCP exciton couples with a fully RCP photon, resulting in a full range of DCP values, i.e., *ρ*_*c*_ from −1 to 1, at the same exciton-polariton band and achiral polaritons at the anticrossing point. The LCP and RCP intensity ratio of polaritons is the sum of that for both the exciton and the photon. In this case, the ratio is 1:0 for the exciton and 0:1 for the photon, leading to an equal intensity of LCP and RCP, thus an achiral response. In Figure 1(f), the photon has a DCP of $\rho_{c}^{p}=0.5$ , with a 3:1 intensity ratio of LCP to RCP. When this photon strongly couples with a fully RCP exciton, the DCP of the LP and UP modes at the anticrossing point is the average of the DCPs of the uncoupled exciton and photon, i.e., $\left(\rho_{c}^{e}+\rho_{c}^{p}\right)/2$ .

**Figure 2:**
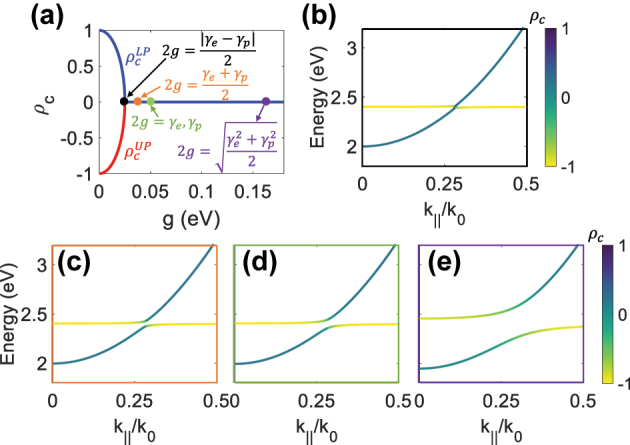
(color online). New criterion of chiral strong light–matter interaction. (a) The DCP values of LP $\rho_{c}^{\text{LP}}$ and UP $\rho_{c}^{\text{UP}}$ as a function of coupling strength *g* at the anticrossing point for an exciton with $\rho_{c}^{e}=-1$ coupled with a photon with $\rho_{c}^{p}=1$ . (b)–(e) Predicted energies of the LP and UP bands in dispersion, with their DCP *ρ*_*c*_ color coded for *g* indicated in (a) as black, orange, green, and purple dots, respectively. Exciton linewidth *γ*_*e*_ = 0.1 eV and photon linewidth *γ*_*p*_ = 0.1 eV.

Strong coupling and the exciton-polariton formation occur when $2g>\frac{\left|\gamma_{e}-\gamma_{p}\right|}{2}$ . Our theoretical model shows the DCP of LP and UP with visible changes in the absence of visible energy splitting. For instance, Figure 2(a) demonstrates an exciton with $\rho_{c}^{e}=-1$ coupled with a photon with $\rho_{c}^{p}=1$ results in zero DCP at the anticrossing point when $2g>\frac{\left|\gamma_{e}-\gamma_{p}\right|}{2}$ . In Figure 2(b), the predicted energies show small splitting; however, notable DCP changes are observed. Energy splitting is only visible with larger *g* (orange, green, and purple dots in Figure 2(a)), as shown in Figure 2(c)–(e). Therefore, our model has introduced a new indicator for chiral strong coupling.

**Figure 3:**
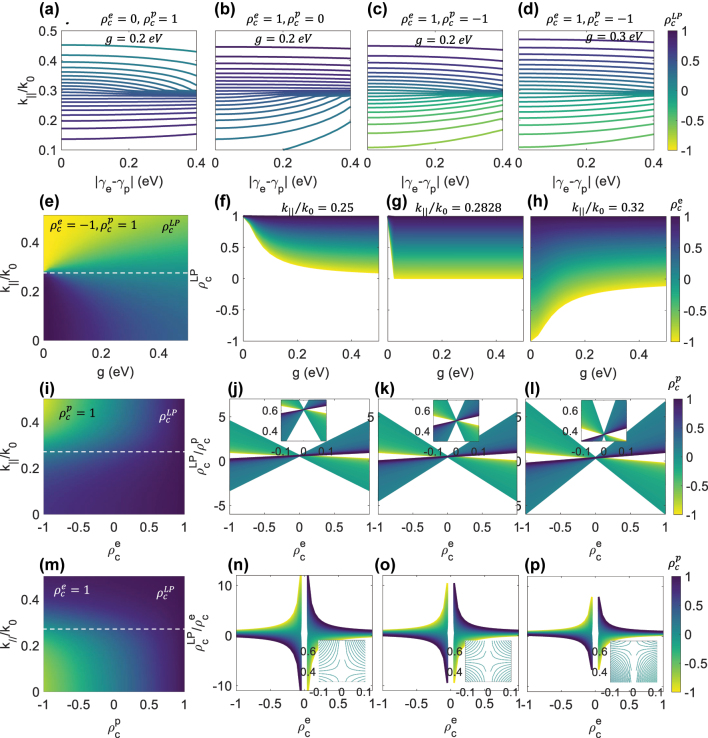
(color online). Contour plots of the LP DCP $\left(\rho_{c}^{LP}\right)$ with the decay rate difference between the exciton and photon (|*γ*_*e*_ − *γ*_*p*_| (eV)). The plots represent the following cases: (a) $\rho_{c}^{e}=0,\;\rho_{c}^{p}=1$ and *g* = 0.2 eV; (b) $\rho_{c}^{e}=1,\;\rho_{c}^{p}=0$ and *g* = 0.2 eV; (c) $\rho_{c}^{e}=1,\;\rho_{c}^{p}=-1$ and *g* = 0.2 eV; (d) $\rho_{c}^{e}=1,\;\rho_{c}^{p}=-1$ and *g* = 0.3 eV. (e) The LP DCP $\left(\rho_{c}^{\text{LP}}\right)$ as a function of coupling strength *g* between a fully RCP exciton $\rho_{c}^{e}=-1$ and a fully LCP photon $\rho_{c}^{p}=1$ . The LP DCP $\left(\rho_{c}^{\text{LP}}\right)$ as a function of *g* between a fully LCP photon $\left(\rho_{c}^{p}=1\right)$ and an exciton with $\rho_{c}^{e}$ in color coded for (f) *k*_‖_/*k*_0_ = 0.25, (g) anticrossing point *k*_‖_/*k*_0_ = 0.2828, and (h) *k*_‖_/*k*_0_ = 0.32. (i) The LP DCP $\left(\rho_{c}^{\text{LP}}\right)$ dispersion as a function of the uncoupled exciton DCP $\rho_{c}^{e}$ under strong coupling with a fully LCP photon at a coupling strength of 0.2 eV. The DCP inheritance of the LP mode from the photon DCP is illustrated for (j) *k*_‖_/*k*_0_ = 0.25, (k) anticrossing point *k*_‖_/*k*_0_ = 0.2828, and (l) *k*_‖_/*k*_0_ = 0.32. (m) The LP DCP dispersion $\left(\rho_{c}^{\text{LP}}\right)$ as a function of the uncoupled photon DCP $\rho_{c}^{p}$ under strong coupling with a fully LCP exciton at a coupling strength of 0.2 eV. The DCP inheritance of the LP mode from the exciton DCP is illustrated for (n) *k*_‖_/*k*_0_ = 0.25, (o) anticrossing point *k*_‖_/*k*_0_ = 0.2828, and (p) *k*_‖_/*k*_0_ = 0.32. The dashed lines in (e), (i), and (m) indicate the anticrossing point.

The criterion for strong coupling is 2*g* > |*γ*_*e*_ − *γ*_*p*_|/2, indicating that the difference in decay rates between the exciton and the photon plays a critical role in their interaction. In Figure 3, we show how the decay rate difference affects the DCP of the LP mode across the in-plane wave vector dispersion. Since the UP mode behaves similarly, we refer to Figure S1(a)–(d) in Supplementary Material S1 for those results. Figure 3(a) shows the LP DCP inherited from a fully LCP photon. The DCP remains largely unaffected by the decay rate difference until the photon couples with the exciton at *k*_‖_/*k*_0_ = 0.2828. Beyond the anticrossing point, the LP DCP rapidly decreases as |*γ*_*e*_ − *γ*_*p*_| increases due to the achiral exciton fraction in the LP mode. In Figure 3(b), when a fully LCP exciton couples with an achiral photon, the LP DCP inherited from the exciton stays largely unchanged for larger wave vectors, even with significant decay rate differences. For smaller wave vectors, the DCP decreases as the decay rate difference increases. Figure 3(c) shows the case where the LP mode inherits DCP from both the exciton and photon. In this case, the DCP returns to the values of the photon or the exciton at wave vectors smaller or larger than the anticrossing point, with the transition accelerating as |*γ*_*e*_ − *γ*_*p*_| increases. However, as shown by comparing Figure 3(c) and (d), this recovery is slowed down by an increase in the coupling strength *g*.

Next, we examine the effects of coupling strength *g* on the LP DCP formed by coupling a fully LCP photon with a fully RCP exciton, as shown in Figure 3(e). When no strong coupling occurs (*g* = 0), the DCP follows the photon’s DCP for wave vectors smaller than the anticrossing point (dashed line) and the exciton’s DCP for larger wave vectors. As *g* increases, the photon and exciton interact more strongly, forming a hybrid LCP and RCP LP mode. At the anticrossing point (*k*_‖_/*k*_0_ = 0.2828), the LP mode inherits equal chirality from the LCP photon and the RCP exciton, resulting in an achiral LP mode with equal LCP and RCP intensities. Figure 3(g) shows the LP DCP at the anticrossing point as a function of *g* for a fully LCP photon and an exciton with $\rho_{c}^{e}$ varying from −1 to 1. The LP DCP is independent of *g* and equals $\left(1+\rho_{c}^{e}\right)/2$ . In Figure 3(f), at *k*_‖_/*k*_0_ = 0.25, the LP DCP starts with the photon’s DCP $\left(\rho_{c}^{p}=1\right)$ and decreases as the exciton becomes more RCP and coupling strength increases. In Figure 3(h), at *k*_‖_/*k*_0_ = 0.32, the LP DCP starts with the exciton’s DCP $\left(\rho_{c}^{e}\right)$ and increases with stronger coupling to the LCP photon (similar results for the UP DCP results are shown in Figure S1(e)–(h) in Supplementary Material S1).

In Figure 3(i), we show the LP DCP formed by coupling a fully LCP photon with an exciton $\left(\rho_{c}^{e}\right)$ . The LP DCP depends more on $\rho_{c}^{p}$ for *k*_‖_/*k*_0_ < 0.2828, where the LP mode is closer to the photon, and more on $\rho_{c}^{e}$ for *k*_‖_/*k*_0_ > 0.2828, where the LP mode is closer to the exciton. At the anticrossing point, the contributions from both the photon and exciton are equal. This is also shown in Figure 3(m), which displays the LP DCP formed by coupling a fully LCP exciton with a photon $\left(\rho_{c}^{p}\right)$ .

Figure 3(j)–(l) show the LP DCP normalized to the uncoupled photon DCP $\left(\rho_{c}^{LP}/\rho_{c}^{p}\right)$ at *k*_‖_/*k*_0_ = 0.25, 0.2828 and 0.32, respectively. At *k*_‖_/*k*_0_ = 0.25, $\rho_{c}^{LP}/\rho_{c}^{p}$ is more compact, indicating a greater dependence on the photon DCP. In the inset of Figure 3(j), the ratio $\rho_{c}^{\text{LP}}/\rho_{c}^{p}$ is approximately 0.6 for the coupling systems, where $\rho_{c}^{p}$ is represented by color coding, and the exciton has $\rho_{c}^{e}=0$ . This indicates that the photon fraction in the LP mode exceeds that of the exciton. In the inset of Figure 3(l), the ratio is about 0.36 for $\rho_{c}^{e}=0$ , indicating that the photon fraction is less than the exciton’s. At the anticrossing point, shown in the inset of Figure 3(k), the ratio is 0.5.

Similarly, Figure 3(n)–(p) show the LP DCP normalized to the uncoupled exciton DCP $\left(\rho_{c}^{\text{LP}}/\rho_{c}^{e}\right)$ at *k*_‖_/*k*_0_ = 0.25, 0.2828 and 0.32, respectively. $\rho_{c}^{\text{LP}}/\rho_{c}^{e}$ is more compact at *k*_‖_/*k*_0_ = 0.32, indicating a greater dependence on the exciton DCP. $\rho_{c}^{\text{LP}}/\rho_{c}^{e}$ diverges for $\rho_{c}^{e}=0$ . This ratio is 0.5 at the anticrossing point for all $\rho_{c}^{e}$ and $\rho_{c}^{p}=0$ , as shown in the inset of Figure 3(o). $\rho_{c}^{\text{LP}}/\rho_{c}^{e}$ shows a smaller value of 0.4 at *k*_‖_/*k*_0_ = 0.25, and a larger value of 0.63 at *k*_‖_/*k*_0_ = 0.32 for all $\rho_{c}^{e}$ and $\rho_{c}^{p}=0$ , as shown in the insets of Figure 3(n) and (p), respectively. Similar results for the UP DCP are shown in Figure S1(i)–(p) in Supplementary Material).

**Figure 4:**
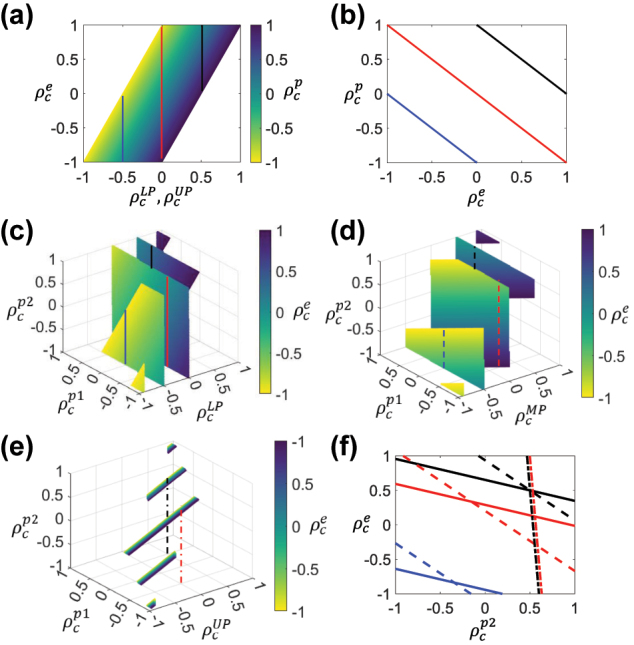
Inverse design the exciton-polaritons with precise chirality (color online). (a) The LP and UP DCP for a two coupled harmonic oscillator model at anticrossing point can be inverse designed by strong coupling of an exciton with DCP $\left(\rho_{c}^{e}\right)$ and a photon with DCP $\left(\rho_{c}^{p}\right)$ . (b) The exciton DCP $\rho_{c}^{e}$ versus photon DCP $\rho_{c}^{p}$ from (a) used to design exciton-polaritons with DCP values of −0.5 (blue), 0 (red), and 0.5 (black) at the anticrossing point. For a three coupled harmonic oscillator model with two photons and an exciton, the LP DCP $\left(\rho_{c}^{\text{LP}}\right)$ (c), middle-polariton (MP) DCP $\left(\rho_{c}^{\text{MP}}\right)$ (d), and UP DCP $\left(\rho_{c}^{\text{UP}}\right)$ (e) at anticrossing point can be inverse designed by strong coupling of an exciton with DCP $\left(\rho_{c}^{e}\right)$ , photon 1 with DCP $\left(\rho_{c}^{p1}\right)$ and photon 2 with DCP $\left(\rho_{c}^{p2}\right)$ . The coupling strength for both coupled harmonic oscillator model is 0.2 eV. (f) $\rho_{c}^{e}$ versus $\rho_{c}^{p2}$ from (c)–(e) used to design of LP (solid), MP (dashed), and UP (dotted) with DCP of −0.5 (blue), 0 (red), and 0.5 (black) at the anticrossing point.

The proposed general analytical model provides insights into the mechanisms driving chirality in exciton-polaritons within strongly coupled systems. Importantly, this model enables the inverse engineering of exciton-polariton bands with tailored chirality in their dispersion for coupled harmonic oscillator models. This indicates that the DCP values of the exciton-polariton states in the in-plane wave vector dispersion can be precisely tuned by specific DCP values of the uncoupled exciton and photon, as well as their coupling strength. As shown in Figure 4(a), in a two-coupled harmonic oscillator model, the LP and UP DCP values vary systematically with the DCPs of their uncoupled components at the anticrossing point; for example, achieving an achiral polariton DCP $\rho_{c}^{\text{LP/UP}}=0$ requires the exciton and photon DCPs to be opposites $\rho_{c}^{p}=-\rho_{c}^{e}$ , as shown by the red line in Figure 4(b). Conversely, a DCP $\rho_{c}^{\text{LP/UP}}=0.5$ is achieved by coupling a fully RCP exciton $\rho_{c}^{e}=1$ with an achiral photon $\rho_{c}^{p}=0$ , as illustrated by the black line in Figure 4(b).

Furthermore, our model is extendable to systems with three or more coupled harmonic oscillator models, offering flexibility in designing exciton-polaritons with complex chiral characteristics. Figure 4(c)–(e) illustrate the dependence of LP, middle polariton (MP), and UP DCP values on the uncoupled exciton and photons DCPs in a three-coupled harmonic oscillator model with two photons and an exciton. The Hamiltonian for this system is given by:

(9) $$H=\left[\begin{array}{ccc} E_{e}-i\frac{\gamma_{e}}{2} & g_{1} & g_{2}\\ g_{1} & E_{p1}-i\frac{\gamma_{p1}}{2} & 0\\ g_{2} & 0 & E_{p2}-i\frac{\gamma_{p2}}{2} \end{array}\right].$$

In this configuration, exciton and photon energies were set to *E*_*e*_ = *E*_*p*1_ = 2.4 eV, *E*_*p*2_ = 2.5 eV, respectively. The linewidths are *γ*_*e*_ = 0.12 eV, *γ*_*p*1_ = *γ*_*p*2_ = 0.16 eV and the coupling strengths are *g*_1_ = *g*_2_ = 0.2 eV. The DCPs of the exciton-polariton states vary in response to the DCPs of exciton and photon, with the UP DCP showing a stronger dependence on the DCP of the second photon due to its energy being farther from the anticrossing point.

In Figure 4(f), we present the relationship between $\rho_{c}^{e}$ and $\rho_{c}^{p}$ based on data in Figure 4(c)–(e). This relationship facilitates the design of LP, MP, and UP with DCP values of −0.5 (blue lines), 0 (red lines), and 0.5 (black lines) at the anticrossing point. For example, an achiral LP can be obtained by strongly coupling photon 1 with $\rho_{c}^{p1}=-0.5$ , photon 2 with $\rho_{c}^{p2}=0$ , and an exciton with $\rho_{c}^{e}=0.2899$ (red line in Figure 4(f)). A UP with $\rho_{c}^{\text{UP}}=-0.5$ cannot be achieved by setting $\rho_{c}^{p1}=0$ for photon 1 (no dotted blue line in Figure 4(f)). Achiral UP with $\rho_{c}^{\text{UP}}=0$ are achievable by setting $\rho_{c}^{p1}=-0.5$ for photon 1, $\rho_{c}^{p2}$ between 0.63 and 0.5 for photon 2, and $\rho_{c}^{e}$ between −1 and 1 for the exciton (red line in Figure 4(f)).

Our model can be extended to include dynamic effects such as spin or polarization relaxation, resulting in time-dependent variations in the exciton-polaritons’ DCP. By introducing a time-dependent coupling strength *g*(*t*), we can predict the evolution of DCP during spin relaxation, aligning our theoretical predictions with experimental studies of cavity polariton spin dynamics in quantum microcavities [47], [48].

## 4 Simulation of DCP of exciton-polaritons in topological photonic crystals

**Figure 5:**
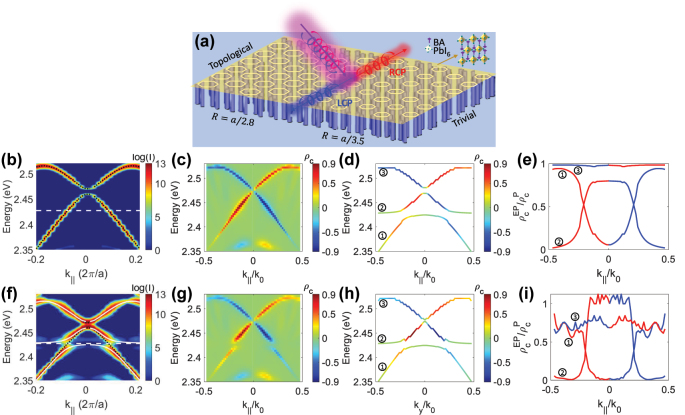
Simulation of DCP of exciton-polaritons in topological photonic crystals (color online). (a) Schematic of the strong coupling system: a 2D halide perovskite exciton strongly coupled to topological edge states formed at the boundary between shrunk (*R* = *a*/3.5) and expanded (*R* = *a*/2.8) hexagonal Si_3_N_4_ photonic lattices. (b) Simulated energy band structure of the topological edge states in photonic crystals, with a lattice constant *a* = 0.4 µm, Si_3_N_4_ pillar height *h* = 1 µm, and diameter *d* = 0.08 µm. The white dashed line marks the exciton energy of 2D halide perovskite, and black dashed curves represent edge state energies. (c) Simulated DCP values for the topological edge states. (d) Calculated exciton-polaritons energies and their DCP values by using a three-coupled harmonic oscillator model with coupling strength *g* = 24 meV and $\rho_{c}^{e}=0$ . (e) Calculated DCP inheritance rate from topological edge states to exciton-polariton bands. (f) Energy band structure of the strong coupling system. The fits to the LP and UP bands are obtained with a three-coupled harmonic oscillator model that describes the interaction between the topological edge states (black dashed curves) and the exciton of 2D halide perovskite (white dashed line), are given as white-solid curves. (g) Simulated DCP *ρ*_*c*_ for the strong coupling system. (h) Energies of the exciton-polaritons extracted from (g), with the DCP values color coded into the bands. (i) Simulated DCP inheritance ratio from topological edge states to exciton-polariton bands.

To validate our analytical model, we performed 3D FDTD simulations to design a superlattice of Si_3_N_4_ dielectric cylinders arranged in an expanded and shrunken honeycomb pattern. Additional simulation details of the photonic crystals and the strong coupling system are provided in Supplementary Material S2 and S3. The simulated energy band structure of the superlattice is shown in Figure 5(b), which displays a pair of topological edge states. Placing the 2D halide perovskite layer on the photonic crystals (Figure 5(a)) induced strong coupling, resulting in two split modes (Figure 5(f)): one at a lower energy and one at a higher energy than the exciton (*E*_*e*_ = 2.427 eV, marked by the white-dashed line in Figure 5(b)). By fitting the peak energies of these bands using a three-coupled harmonic oscillator model, we determined a strong coupling strength of 24 meV.

To determine the DCP of the topological edge states in the photonic crystals, we used the Lorentz reciprocity principle [49], which allows us to calculate emitted light by evaluating field enhancements under circularly polarized (CP) incident light. This approach lets us switch between the source and detector of electromagnetic fields, enabling the calculation of far-field emitted power and polarization from randomly placed dipoles by assessing field enhancements at their locations (see Supplementary Material S4).

In the simulations, CP plane waves are directed onto the photonic crystal from angles *θ* = −30° to 30° and polarizations *ϕ* = −180° to 180°. The field enhancement factor for each angle and polarization is calculated by integrating the total electric field intensity within the 2D halide perovskite region and normalizing it to the intensity in free space, i.e.,

(10) $$I\left(\lambda \right)=\frac{\underset{V}{∭}|E\left(x,y,z,\lambda \right){|}^{2}\text{d}x\text{d}y\text{d}z}{\underset{V}{∭}|E_{\mathrm{ref}}\left(x,y,z,\lambda \right){|}^{2}\text{d}x\text{d}y\text{d}z},$$

where *E*(*x*, *y*, *z*, *λ*) is the electric field with the photonic crystals present, *E*_ref_(*x*, *y*, *z*, *λ*) is the field without the photonic crystals, and the integrals are evaluated over the unit cell area *V*.

The simulated field enhancements for the topological edge states and the exciton-polaritons under LCP and RCP light, i.e., *I*_LCP_ and *I*_RCP_, are shown in Figure S5 in Supplementary Material S4. Figure 5(c) shows the resulting DCP *ρ*_*c*_ across the energy spectra, with reciprocity-based calculations highlighting a chiral response from the topological edge states, achieving a high DCP (*ρ*_*c*_ = ∓0.847) at *k*_*y*_ = ±0.1145 *k*_0_.

To examine DCP transfer from topological edge photons to hybrid exciton-polaritons under strong coupling, we used a three-coupled harmonic oscillator model to calculate the exciton-polaritons energies and their DCP values as shown in Figure 5(d). Figure 5(e) shows that the inheritance $\rho_{c}^{\text{EP}}/\rho_{c}^{p}$ is about 0.5 for bands ① and ② at the strong anticrossing point (*k*_*y*_/*k*_0_ = ±0.2114), indicating equal photon contribution. For the highest energy band (③), the ratio $\rho_{c}^{\text{EP}}/\rho_{c}^{p}$ approaches 1, indicating that this band is mostly photon-based, as it lies farther from the exciton energy. To compare the model-calculated DCP of exciton-polaritons, we simulated the DCP of the strong coupling system, shown in Figure 5(f), where chiral exciton-polaritons appear as blue and red regions. By extracting the energies and DCP peak values in Figure 5(g), we derived the energy bands and DCPs of the exciton-polaritons in Figure 5(h). Figure 5(i) shows the DCP inheritance for bands ①, ②, and ③, consistent overall with the theoretical model results in Figure 5(e). However, the highest energy band, influenced more by the achiral exciton, inherits only about 0.7 of the photon’s chirality. At the anticrossing point (*k*_*y*_/*k*_0_ = ±0.2114), the lower two energy bands equally inherit the DCP of topological edge states, resulting in an inheritance $\rho_{c}^{\text{EP}}/\rho_{c}^{p}$ of 0.4. Additionally, our theoretical model accurately predicted the transmission dissymmetry *g*_trans_ for lower and upper polaritons in chiral (R-MBA)PbI_3_ and (R-MBA)_2_PbI_4_ films within an FP cavity, closely matching the experimental data (see Supplementary Material S5).

## 5 Conclusions

In conclusion, we have developed a generally applicable analytical model that provides deep insight into the mechanisms responsible for chirality in exciton-polaritons. Built upon the framework of coupled harmonic oscillators, this model offers reliable predictions of the DCP of exciton-polaritons and introduces a new criterion for chiral strong coupling upon their formation, making it a valuable tool to aid the simulation and experimental approaches in the field. The model captures three key behaviors that are critical for understanding and designing chiral exciton-polaritons: (a) at the anticrossing point, the DCP of exciton-polaritons results from an equal contribution of the DCPs from both the exciton and photon components. This finding highlights the balanced interaction between these two constituents at the strong anticrossing point. (b) As the system moves away from the anticrossing point, the DCP of exciton-polaritons transitions toward the dominant component, whether it be the exciton or the photon. Notably, these transitions occur more gradually as the coupling strength increases, providing insight into how varying coupling conditions influence the chirality of the system. And (c) the model predicts that a single exciton-polariton band can exhibit a full range of DCP values, from −1 to +1, which enables the design of exciton-polaritons with tailored chirality. Moreover, our model may be applied for designing the chirality of complex systems, for instance, systems involving multiple photons and excitons, exciton-phonon-polaritons, and exciton-plasmon-polaritons.

To validate our model, we conducted simulations of a photonic crystal superlattice featuring topological edge states, which are strongly coupled to a 2D halide perovskite exciton. Using the reciprocity principle, we observed a chiral response in the topological edge state bands, which was effectively transferred to the exciton-polariton bands. The simulated DCP values demonstrated good agreement with the model’s predictions by a three-coupled harmonic oscillator model. Our study enhances the understanding of chiral inheritance in exciton-polaritons and reveals how chirality is transferred and maintained in these hybrid systems. Additionally, our analytical model offers a practical tool for the inverse design of exciton-polaritons with precise chirality. Using this model, researchers can predict and tailor the chiral properties of exciton-polaritons for specific applications, advancing the development of chiral optoelectronic devices.

## Supplementary Material

Supplementary Material Details
