# Supplementary material for: A general model for designing the chirality of exciton-polaritons
Source: Nanophotonics. 2025 Feb 3;14(3):407–16. doi: 10.1515/nanoph-2024-0662 (PMC11831404; doi:10.1515/nanoph-2024-0662)
Supplement: Supplementary file 1 — Supplementary Material Details [file j_nanoph-2024-0662_suppl_001.pdf]

Research Article

Ping Bai and Siying Peng*

Supplementary Material:A general model for designing the chirality of exciton-polaritons

S1 Numerical analysis of DCP in upper polariton (UP)

In Fig. S1, we firstly show the decay rate difference affects the DCP of the UP mode across the in-plane wave vector dispersion. Fig. S1 (a) shows the UP DCP inherited from a fully LCP photon. The UP DCP inherited from the exciton stays largely unchanged for larger wave vectors, even with significant decay rate differences. For smaller wave vectors, the DCP decreases as the decay rate difference $|\gamma_e - \gamma_p|$ increases. In Fig. S1 (b), when a fully LCP exciton couples with an achiral photon, the DCP remains largely unaffected by the decay rate difference until the photon couples with the exciton at $k_{||}/k_0 = 0.2828$. Beyond the anti-crossing point, the UP DCP rapidly decreases as $|\gamma_e - \gamma_p|$ increases due to the achiral exciton fraction in the UP mode. Fig. S1 (c) shows the case where the UP mode inherits DCP from both the exciton and photon. In this case, the DCP returns to the values of the photon or exciton at wave vectors smaller or larger than the anti-crossing point, with the transition accelerating as $|\gamma_e - \gamma_p|$ increases. However, as shown by comparing Figs. S1 (c) and (d), this recovery is slowed down by an increase in the coupling strength g.

Next, we examine the effects of coupling strength g on the UP DCP formed by coupling a fully LCP photon with a fully RCP exciton, as shown in Fig. S1 (e). When no strong coupling occurs ($g = 0$), the DCP follows the exciton's DCP for wave vectors smaller than the anti-crossing point (dashed line) and the photon's DCP for larger wave vectors. As g increases, the photon and exciton interact more strongly, forming a hybrid LCP and RCP UP mode. At the anti-crossing point ($k_{||}/k_0 = 0.2828$), the UP mode inherits equal chirality from the LCP photon and RCP exciton, resulting in an achiral UP mode with equal LCP and RCP intensities. Fig. S1 (g) shows the UP DCP at the anti-crossing point as a function of g for a fully LCP photon and an exciton with ρ_c^e varying from -1 to 1. The UP DCP is independent of g and equals $(1 + \rho_c^e)/2$. In Fig. S1 (f), at $k_{||}/k_0 = 0.25$, the UP DCP starts with the exciton's DCP (ρ_c^e) and increases with stronger coupling to the LCP photon. In Fig. S1 (h), at $k_{||}/k_0 = 0.32$, the UP DCP starts with the photon's DCP ($\rho_c^p = 1$) and decreases as the exciton becomes more RCP and coupling strength increases.

In Fig. S1 (i), we show the UP DCP formed by coupling a fully LCP photon with an exciton (ρ_c^e). The UP DCP depends more on ρ_c^e for $k_{||}/k_0 < 0.2828$, where the UP mode is closer to the exciton, and more on LCP photon for $k_{||}/k_0 > 0.2828$, where the UP mode is closer to the photon. At the anti-crossing point, the contributions from both the photon and exciton are equal. This is also shown in Fig. S1 (m), which displays the UP DCP formed by coupling a fully LCP exciton with a photon (ρ_c^p).

Ping Bai, School of Engineering, Westlake University, Hangzhou, Zhejiang 310030, China, baiping@westlake.edu.cn; https://orcid.org/0000-0002-7004-6501
***Corresponding author: Siying Peng,** School of Engineering and Research Center for Industries of the Future, Westlake University, Hangzhou,Zhejiang 310030, China, pengsiying@westlake.edu.cn; https://orcid.org/0000-0002-1541-0278

Figs. S1 (j), (k) and (l) show the UP DCP normalized to the uncoupled photon DCP (ρ_c^{UP}/ρ_c^p) at $k_{||}/k_0 = 0.25, 0.2828$ and 0.32, respectively. The ratio is more compact at $k_{||}/k_0 = 0.32$, indicating greater dependence on the photon DCP. In the inset of Fig. S1 (j), the ratio ρ_c^{UP}/ρ_c^p is 0.36 for the coupling systems, where ρ_c^p is represented by color coding, and the exciton has $\rho_c^e = 0$. This indicates the photon fraction is less than that of exciton. In the inset of Fig. S1 (l), the ratio is 0.6 for $\rho_c^e = 0$, indicating that the photon fraction in the UP mode exceeds that of the exciton. At the anti-crossing point, shown in the inset of Fig. S1 (k), the ratio is 0.5.

Similarly, Figs. S1 (n), (o) and (p) show the UP DCP normalized to the uncoupled exciton DCP (ρ_c^{UP}/ρ_c^e) at $k_{||}/k_0 = 0.25, 0.2828$ and 0.32, respectively. ρ_c^{UP}/ρ_c^e is more compact at $k_{||}/k_0 = 0.25$, indicating greater dependence on the exciton DCP. ρ_c^{UP}/ρ_c^e diverges for $\rho_c^e = 0$. This ratio is 0.5 at the anti-crossing point for all ρ_c^e and $\rho_c^p = 0$, as shown in the inset of Fig. S1 (o). ρ_c^{UP}/ρ_c^e shows a smaller value of 0.4 at $k_{||}/k_0 = 0.32$, and a larger value of 0.63 at $k_{||}/k_0 = 0.25$ for all ρ_c^e and $\rho_c^p = 0$, as shown in the inset of Figs. S1 (n) and (p), respectively.

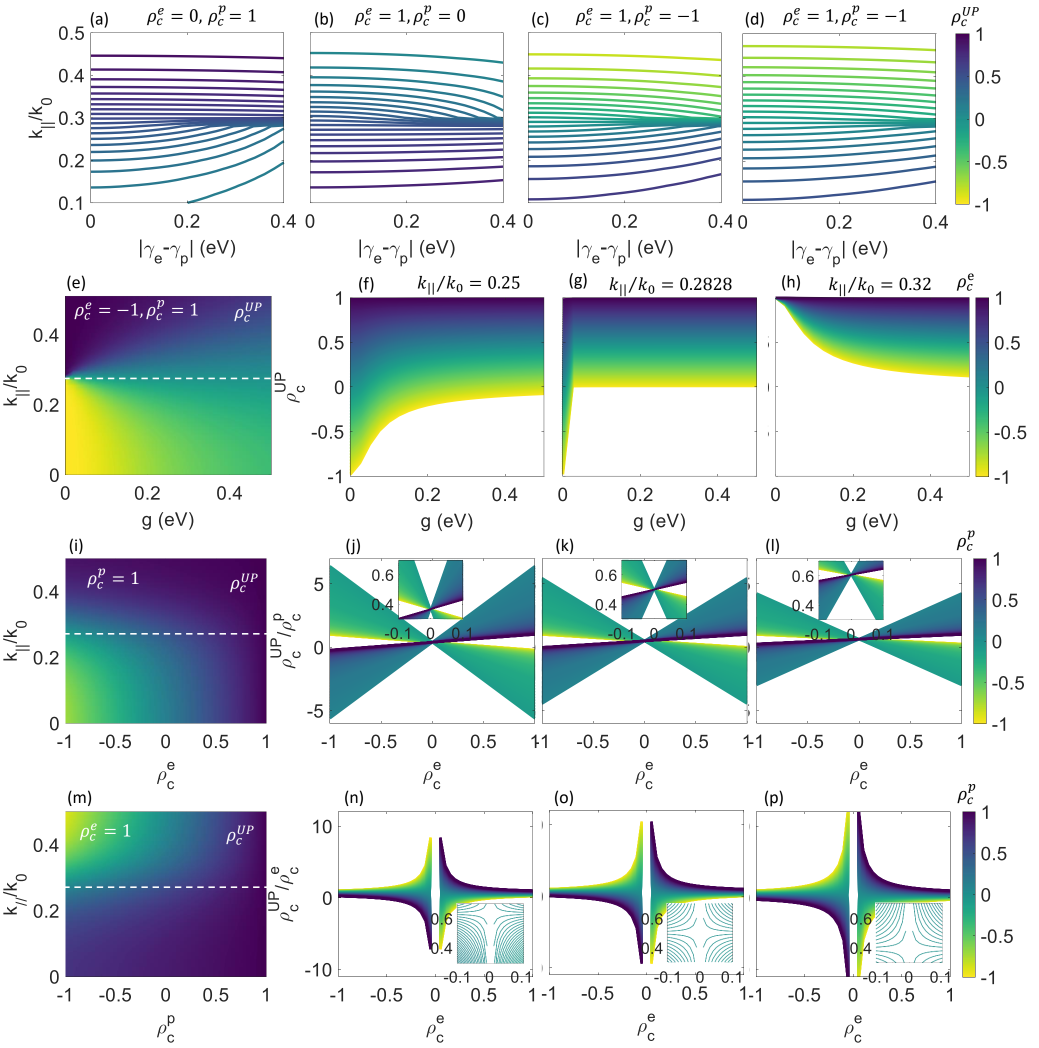

Fig. S1: (color online). Contour plots of the UP DCP (ρ_c^{UP}) in dispersion with the decay rate difference between the exciton and photon ($|\gamma_e - \gamma_p|$ (eV)). The plots represent the following cases: (a) $\rho_c^e = 0$, $\rho_c^p = 1$ and $g = 0.2eV$; (b) $\rho_c^e = 1$, $\rho_c^p = 0$ and $g = 0.2$ eV; (c) $\rho_c^e = 1$, $\rho_c^p = -1$ and $g = 0.2$ eV; (d) $\rho_c^e = 1$, $\rho_c^p = -1$ and $g = 0.3$ eV. (e) The UP DCP (ρ_c^{UP}) as a function of coupling strength g between a fully RCP exciton $\rho_c^e = -1$ and a fully LCP photon ($\rho_c^p = 1$). The UP DCP (ρ_c^{UP}) as a function of g between a fully LCP photon ($\rho_c^p = 1$) and an exciton with ρ_c^e in color coded for (f) $k_{||}/k_0 = 0.25$, (g) anti-crossing point $k_{||}/k_0 = 0.2828$ and (h) $k_{||}/k_0 = 0.32$. (i) The UP DCP (ρ_c^{UP}) dispersion as a function of the uncoupled exciton DCP ρ_c^e under strong coupling with a fully LCP photon at a coupling strength of 0.2 eV. The DCP inheritance of the UP mode from the photon DCP is illustrated for (j) $k_{||}/k_0 = 0.25$, (k) anti-crossing point $k_{||}/k_0 = 0.2828$ and (l) $k_{||}/k_0 = 0.32$. (m) The UP DCP dispersion (ρ_c^{UP}) as a function of the uncoupled photon DCP ρ_c^p under strong coupling with a fully LCP exciton at a coupling strength of 0.2 eV. The DCP inheritance of the UP mode from the exciton DCP is illustrated for (n) $k_{||}/k_0 = 0.25$, (o) anti-crossing point $k_{||}/k_0 = 0.2828$ and (p) $k_{||}/k_0 = 0.32$. The dashed lines in (e), (i) and (m) indicate the anti-crossing point.

S2 Topological edge states in photonic crystals

We begin by simulating the energy band structure of a three-dimensional (3D) honeycomb lattice composed of Si_3N_4 dielectric cylinders (Fig. S2 (a)) using the finite-difference time-domain (FDTD) method. As shown in Fig. S2 (d), the band dispersion exhibits double degeneracy at Dirac points located at the Γ point, with an energy around 2.5 eV. Next, we deform the 3D honeycomb lattice while maintaining the hexagonal clusters formed by six neighboring cylinders and preserving the C_6 symmetry. For the expanded lattice ($R = a/2.8$) shown in Fig. S2 (b), the previously degenerate photonic band opens, creating a band gap, as illustrated in Fig. S2 (e). In this expanded lattice, enhanced intracell coupling leads to band hybridization due to band winding, making the band gap topologically nontrivial. Furthermore, by shrinking the hexamer unit cell (Fig. S2 (c)) with $R = a/3.5$, the degenerate Dirac point is split. In the shrunk lattice, enhanced intercell coupling results in the separation of the Dirac cones and the formation of a trivial band gap in the energy band structure, as shown in Fig. S2 (f).

Next, we combine the expanded and shrunken lattices to form a super-lattice, creating an interface between them. Simulating the energy band structure reveals a pair of in-gap states in the band dispersion, as shown in Fig. S2 (g). These states correspond to the topological edge states located at the interface between the topologically trivial and non-trivial regions. By examining the real-space distribution of the E_z field at momenta near the Γ point (A and B in Fig. S2 (g) with $k_x = 0.05(2\pi/a)$), we observe that the in-gap states located at the ribbon edges and decay exponentially into bulk as illustrated in Fig. S2 (h). The middle insets of Fig. S2 (h) show that the Poynting vectors exhibit left circularly polarized energy flow at point A, and right circularly polarized energy flow at point B. These resonances are attributed to the topological edge states, characterized by pseudospin-up and pseudospin-down of photons, respectively.

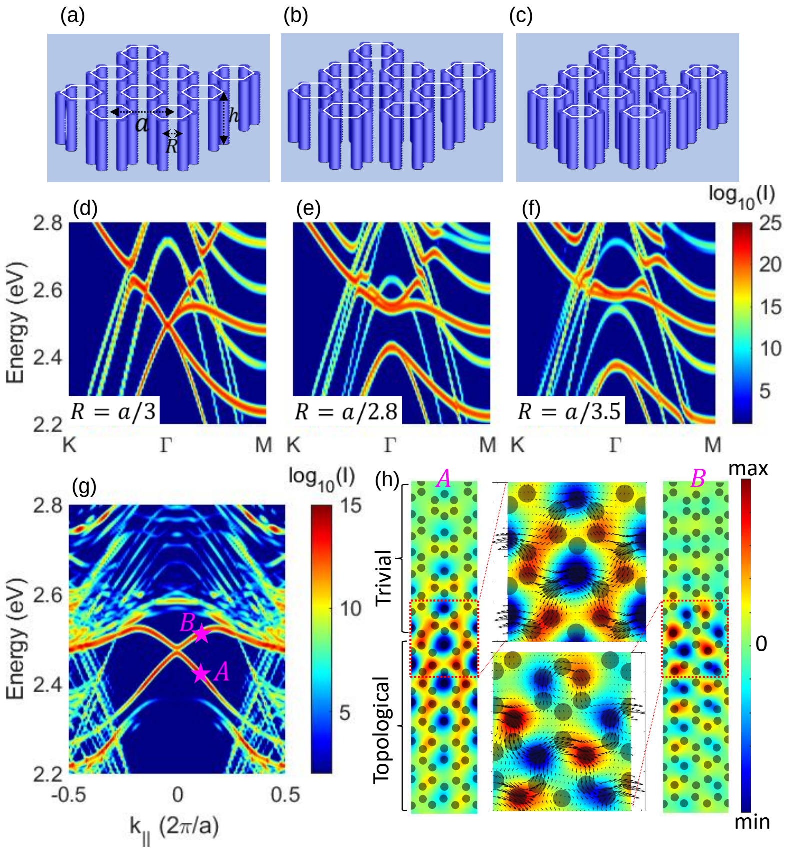

Fig. S2: (color online). Schematic plot of (a) hexagonal photonic honeycomb lattice, (b) expanded lattice and (c) shrunken lattice composed of Si_3N_4 pillars, where a is the lattice constant, R is the minimum distance of the Si_3N_4 pillars in the unit cell. Simulated energy band structure of 3D photonic crystals with $a = 400\ nm$, the height of pillars $h = 1\ \mu m$ and the dimeter $d = 95\ nm$ for (d) hexagonal lattice ($R = a/3$), (e) expanded lattice ($R = a/2.8$) and (f) shrunken lattice ($R = a/3.5$). (g) Simulated energy band structure for edge states between expanded and shrunk hexagonal photonic lattices. (h) Real-space distributions of electric field E_z for edge states at points A and B indicated in (g). The black arrows in the inlarged visions represent the real part of the Poynting vectors projected in the xy-plane.

S3 Strong coupling system: 2D halide perovskite on top of photonic crystals

The topological helical exciton-polariton system we describe is illustrated schematically in Fig. S3 (a). It features a Si_3N_4 photonic cystal that supports topological edge sates at the boundary between a shrunken and an expanded hexagonal photonic lattice. On top of this photonic crystal, a 3 nm layer of 2D Ruddlesden-Popper $(BA)_2PbI_2$ perovskite is placed, where (BA) represents $CH_3(CH_2)_3NH_3$. The optical properties of the 2D halide perovskite, defined by the refractive index n and dielectric loss κ, are shown in Fig. S3 (b). These properties reveal the 2D halide perovskite exciton energy at $2.427eV$ and a background refractive index of 2. The exciton is a flat band in energy structure, as shown in Fig. S3 (c), with full width at half maxima (FWHM) of about 0.02 eV.

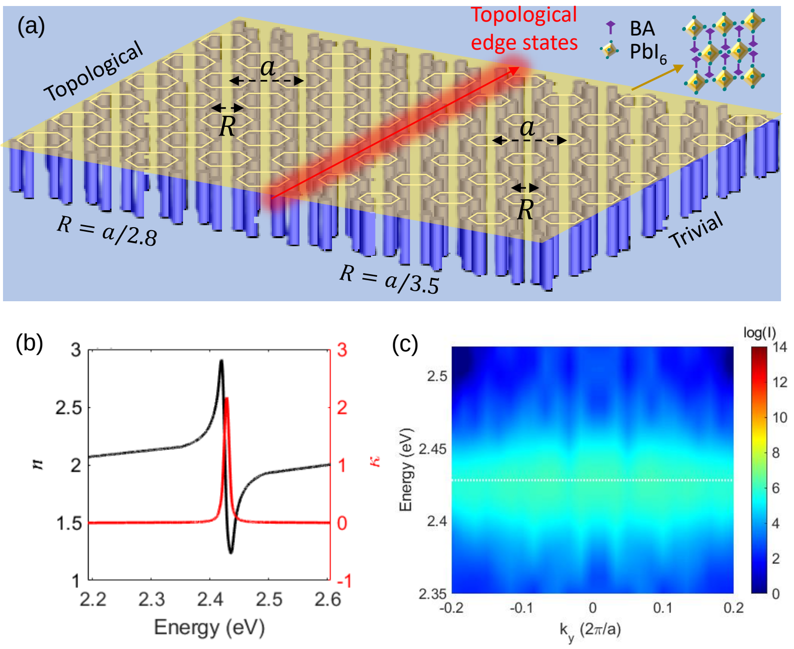

Fig. S3: (color online). (a) Schematic representation of the strong coupling system: 2D halide exciton strongly coupled to the topological edge states formed by the edge between the shrunk ($R = a/3.5$) and expanded ($R = a/2.8$) hexagonal Si_3N_4 photonic lattices. (b) Complex refractive index of 2D halide perovskite. The black curve represents the real component (refractive index) whereas the red curve represents the imaginary component (dielectric loss). (c) Energy band structure of the layer of 2D halide perovskite).

S4 Simulated LCP and RCP field enhancements for topological edge states and exciton-polaritons

According to the Lorentz reciprocity principle, the problem of light emission is converted into a field enhancement calculation under circularly polarized (CP) incident light, as shown in Fig. S4 (a). The reciprocity principle states that the source and detector of electromagnetic fields can be interchanged. Therefore, the far-field emitted power and polarization from an ensemble of randomly positioned and oriented dipoles can be determined by calculating the field enhancements at the dipole positions under incident light.

In our simulations, CP plane waves are incident onto the photonic crystal and the coupling system at different angles and polarizations. Fig. S4 (b) shows the reflectance of the photonic crystal under LCP and RCP light, each incident at an angle of 7°. It's observed that the lower energy band ($E = 2.45\ eV$) corresponds to LCP, while the higher energy band ($E = 2.48\ eV$) corresponds to RCP. The real-space distributions of the electric field component E_z in the 2D halide perovskite region for both bands, at points A and B indicated in Fig. S2 (g), are shown in Figs. S4 (c) and (d). The time-averaged Poynting vectors around the interface between the shrunken and expanded lattices indicate the topological edge state at point A is left circularly polarized, while the state at point B band is right circularly polarized.

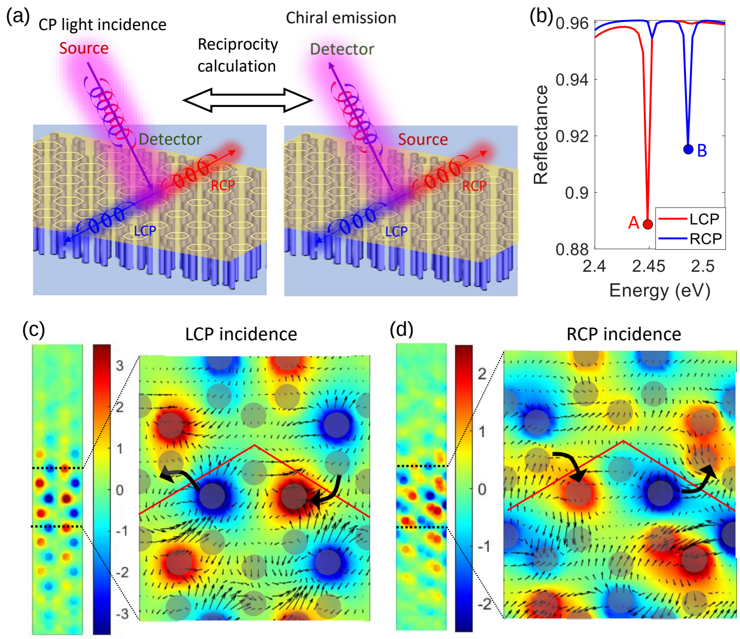

Fig. S4: (color online). (a) Schematic for the reciprocity calculation of chiral emission. The reciprocity principle states that the source and detector of electromagnetic fields can be interchanged. Therefore, chiral emission can be calculated by integrating the field enhancement in the light-emitting medium under circularly polarized incidence. (b) Reflectance spectra of photonic crystal superlattice under the LCP and RCP incidence with 7°. (c) and (d) Real-space distributions of E_z fields at points A ($E = 2.45\ eV$) and B ($E = 2.48\ eV$) indicated in Fig. S4 (b) for LCP and RCP incidence, respectively. Right panels: time-averaged Poynting vectors $\vec{S} = Re[\vec{E} \times \vec{H}^*]/2$ around the interface between shrunk and expanded lattices (solid-red lines). Black arrows indicate the direction of the current.

By integrating the total electric field intensity in the 2D halide perovskite region and normalizing it to the integrated field intensity in empty space, we calculate the field enhancement factor for each incident angle and polarization (or emission enhancement factor for each emission angle and

polarization): I_{LCP} and I_{RCP} for the LCP and RCP incidences, respectively. Since the 2D halide perovskite is achiral, the DCP is defined as $\rho_c = (I_{LCP} - I_{RCP})/(I_{LCP} - I_{RCP})$. Figs. S5 (a) and (b) show the LCP and RCP emission enhancements for the bare supperlattice, respectively, while Fig. S5 Figs. S5 (c) and (d) show the LCP and RCP emission enhancements for the strong coupling system, respectively. At the 2D halide perovskite exciton energy ($E_e = 2.427\ eV$), a dark emission band is observed, resulting from exciton absorption.

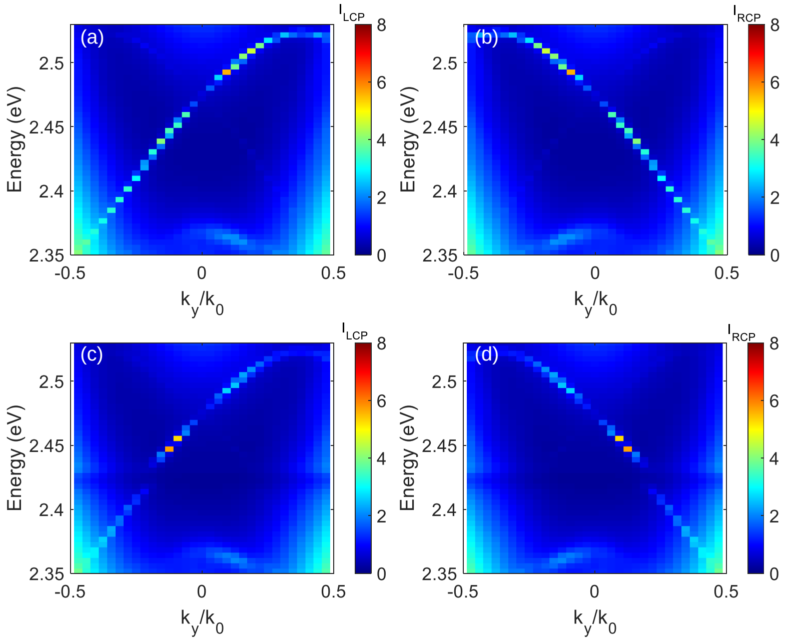

Fig. S5: (color online). (a) LCP and (b) RCP emission enhancements for topological photonic crystals. (c) LCP and (d) RCP emission enhancements for exciton-polaritons.

S5 Verify the theoretical model with chiral perovskite

To validate our analytical model with chiral emitters, we compared it to experimental transmission dissymmetry g_{trans} data of chiral perovskites from the literature [1]. From Fig. 5b in Ref. [1], which presents g_{trans} for (R-MBA)PbI_3 and (R-MBA)$_2PbI_4$ 50:50 wt% films embedded in a FP cavity under normal incident light, we extracted g_{trans} values of $+0.13016\%$ (front-incident) and -0.11898% (back-incident) for exciton X.

Using our model with the following parameters: the (R-MBA)$_2PbI_4$ exciton energy $E_e = 2.48$ eV, cavity energy $E_p = 2.4$ eV, cavity linewidth $\gamma_p = 0.05$ eV, exciton linewidth $\gamma_e = 0.11$ eV, and a Rabi splitting of 0.13 eV, we predicted g_{trans} values for the LP and UP modes. For front normal incidence, the predicted g_{trans} are -0.7376% (LP) and -0.1346% (UP), which closely match the experimental values of -0.71604% (LP) and -0.09091% (UP) shown in Fig. 5b of Ref. [1]. Similarly, for back normal incidence, our predictions of 0.7400% (LP) and 0.1431% (UP) correspond well with the experimental values of 0.7059% (LP) and 0.10325% (UP).

Table S1 compares the experimental transmission dissymmetry g_{trans} values of chiral perovskites and exciton-polaritons in a FP cavity, as reported in the literature [1], with the predicted g_{trans} values for the LP and UP modes obtained using our theoretical model. The close agreement between experimental and predicted values validates the accuracy of our model in describing the behavior of chiral exciton-polaritons.

Tab. S1: Summary of experimental transmission dissymmetry g_{trans} values of chiral perovskites and exciton-polaritons in a FP cavity reported in the literature [1], and the predicated g_{trans} values for the LP and UP modes by our theoretical model.

	g_{trans} of exciton X (Fig. 5b in [1])	incident light	g_{trans} of LP (Fig. 5b in [1])	g_{trans} of UP (Fig. 5b in [1])	g_{trans} of LP (model predict)	g_{trans} of UP (model predict)
Front	0.13016	-1	-0.71604	-0.09091	-0.7376	-0.1346
Back	-0.11898	1	0.7059	0.10325	0.7400	0.1431

References

[1] Z. Wang, C.-C. Lin, K. Murata, *et al.*, "Chiroptical response inversion and enhancement of room-temperature exciton-polaritons using 2d chirality in perovskites," *Advanced Materials*, vol. 35, no. 42, p. 2 303 203, 2023.
